# Efficacy of Wenxin Keli Plus Amiodarone versus Amiodarone Monotherapy in Treating Recent-Onset Atrial Fibrillation

**DOI:** 10.1155/2018/6047271

**Published:** 2018-12-04

**Authors:** Nixiao Zhang, Gary Tse, Shristi Dahal, Yajuan Yang, Mengqi Gong, Calista Zhuo Yi Chan, Enzhao Liu, Gang Xu, Konstantinos P. Letsas, Panagiotis Korantzopoulos, Guangping Li, Tong Liu

**Affiliations:** ^1^Tianjin Key Laboratory of Ionic-Molecular Function of Cardiovascular Disease, Department of Cardiology, Tianjin Institute of Cardiology, Second Hospital of Tianjin Medical University, Tianjin 300211, China; ^2^Department of Medicine and Therapeutics, Chinese University of Hong Kong, Ma Liu Shui, Hong Kong; ^3^Li Ka Shing Institute of Health Sciences, Faculty of Medicine, Chinese University of Hong Kong, Ma Liu Shui, Hong Kong; ^4^School of Health Sciences, University of Manchester, Manchester, UK; ^5^Second Department of Cardiology, Laboratory of Cardiac Electrophysiology, “Evangelismos” General Hospital of Athens, Athens, Greece; ^6^First Department of Cardiology, University of Ioannina Medical School, Stavrou Niarchou-1, 45221 Ioannina, Greece

## Abstract

**Background:**

Use of amiodarone (AMIO) in atrial fibrillation (AF) has significant side effects over prolonged periods. Wenxin Keli (WXKL), a Chinese herb extract, has been shown to be effective in atrial-selective inhibiting peak *I*_Na_ and hence beneficial in treating atrial arrhythmias, including atrial fibrillation. The aim of this randomized controlled trial was to evaluate potential effects of AMIO plus WXKL on conversion rate and time in patients with recent-onset AF.

**Methods:**

A total of 41 patients (71 ± 12 years, 44% male) with recent-onset (<48 h) AF eligible for conversion were randomized to receive either intravenous amiodarone (loading dose 5 mg/kg in 1 hour followed by 50 mg/h; *n*=21) or amiodarone with same dosage plus oral WXKL 18 g thrice daily (*n*=20) for 24 hours.

**Results:**

Conversion rate at 24 hours was of no difference between the two groups (75.0% vs. 81.0%, *P*=0.72); however, conversion time was markedly shorter in the AMIO + WXKL group compared to the AMIO group (291 ± 235 minutes vs. 725 ± 475 minutes, *P*=0.003). There were no serious adverse events during the study.

**Conclusion:**

Administration of amiodarone plus WXKL for recent-onset AF conversion was safe and effective, with faster sinus rhythm restoration compared with amiodarone alone.

## 1. Introduction

Atrial fibrillation (AF), the most common cardiac arrhythmia, is independently responsible for a five-fold increased risk of stroke, a three-fold increased risk of heart failure, and causes significant mortality [1]. By 2030, 14–17 million individuals living in the European Union are predicted to suffer from AF. Restoration and maintenance of sinus rhythm is a major strategy for AF management [2]. The 2012 European Society of Cardiology (ESC) guidelines for the management of AF recommend that amiodarone (AMIO) be used for conversion in patients with ischemic or structural heart disease [2]. However, AMIO has extra-cardiac toxic effects, only moderate efficacy, and a delayed onset of action [1]. By contrast, Wenxin Keli (WXKL), a traditional Chinese medicine composed of Nardostachys, Codonopsis, Notoginseng, Amber, and Rhizoma, is an approved preparation that has demonstrated efficacy and safety in the treatment of several forms of cardiac arrhythmia. In isolated canine, arterially perfused right atrial preparations, Wenxin Keli significantly prolonged effective refractory period (ERP) and induced postrepolarization refractoriness (PRR) despite abbreviating the action potential duration in an atrial-selective manner. It also reduced maximum rate of rise of action potential upstroke, prolonged P wave duration such that sodium channel current (*I*_Na_) dependent parameters were depressed, and prevented induction of persistent AF in 100% of preparations tested [3]. Burashnikov et al. reported that WXKL has anti-AF properties due to its atrial-selective depression of *I*_Na_-dependent parameters in canine-isolated coronary-perfused preparations [3]. The atrial selectivity of WXKL likely contributes to its usefulness for effective management of AF with minimal effects on the ventricular electrophysiology [3–5]. Atrial-selective *I*_Na_ block occurred following trains of pulses elicited over a range of pulse durations and interpulse intervals in canine myocytes. This was due to a combination of the more negative voltage of steady-state inactivation, the less-negative resting membrane potential, and the shorter diastolic intervals in atrial cells at rapid activation rates [6]. When used in combination with AMIO, WXKL has been found to shorten conversion time, decrease the required dosage of AMIO, and prevent adverse drug reactions [7]. The aim of this pilot study was to evaluate the effects of adding WXKL to AMIO on conversion rate and time within 24 hours after administration in patients with recent-onset AF.

## 2. Methods

### 2.1. Study Protocol

This was a single-center, randomized, open-label prospective clinical trial in the Second Hospital of Tianjin Medical University. The study protocol conformed to the ethical guidelines of the 1975 Declaration of Helsinki and the CONSORT recommendations and was approved by the Institutional Committee on Human Research of the Second Hospital of Tianjin Medical University. Agents used in this trial were approved by the State Food and Drug Administration (SFDA) and have become available in the market for the treatment of cardiac arrhythmias. All patients provided their informed consent. Eligible patients, in accordance with the random number table, were divided into two groups (random numbers 1 to 21 for AMIO group, random numbers 22 to 41 for AMIO + WXKL group) and assigned to receive either intravenous AMIO (loading dose 5 mg/kg in 1 hour followed by 50 mg/h) or AMIO with the same dosage plus oral WXKL 18 g three times daily for a maximum of 24 hours. All of the patients received appropriate anticoagulation therapy before and during conversion as per recommended guidelines. After conversion to sinus rhythm, both groups continued to receive AMIO orally 200 mg three times a day for a week, 200 mg twice a day for the next week, and once a day afterwards, or as according to the physician's prescription. Βeta-receptor blockers were allowed in these two groups to achieve rate control. Furthermore, patients underwent careful 24-hour electrocardiogram (ECG) monitoring during the course of the treatment. The trial continued until one or more of the following events were encountered: new onset sustained ventricular tachycardia, ventricular fibrillation, or torsades de pointes; QTc >550 ms; heart rate <40 bpm or symptomatic bradycardia; systolic blood pressure <90 mmHg; acute hyperthyroidism; allergic shock; and severe superficial phlebitis. In cases of AF persisting beyond 24 hours, electrical conversion or radiofrequency ablation was attempted directly (without atrial thrombus) or after 4 weeks of anticoagulation administration.

### 2.2. Patient Selection and Exclusion Criteria

All patients who developed recent-onset (duration <48 hours) AF and presented from February 2016 to December 2016 were eligible for our study. All patients were fit for pharmacological conversion as well as adequate anticoagulation therapy in accordance with guidelines. Exclusion criteria were acute coronary syndrome, cardiogenic shock, atrial flutter, symptomatic bradycardia, history of sick sinus syndrome or atrioventricular block, severe valvular diseases, thyroid disorders, renal failure, and administration of Classes I or III antiarrhythmic drugs within 24 hours before enrolling into the study.

### 2.3. Endpoints and Clinical Parameters

The primary endpoint was the conversion rate of AF to SR within 24 hours in the AMIO group and the AMIO + WXKL group. The secondary endpoint was the conversion time in these two groups within 24 hours. In terms of electrocardiography (ECG), QT interval was measured at baseline, at the time of conversion to SR (success in conversion), or at 24 hours (failure in conversion), and then corrected for heart rate (QTc) using Bazett's formula. Transthoracic echocardiographic (TTE) parameters were recorded at baseline, including left atrial diameter (LAD), left ventricular end diastolic diameter (LVEDD), left ventricular systolic diameter (LVSDD), interventricular septal (IVS) thickness, and left ventricular ejection fraction (LVEF). In addition, biochemical parameters, including serum potassium, serum sodium, serum chlorine, serum creatinine, blood uric acid, and blood nitrogen, were assessed at baseline.

### 2.4. Statistical Analyses

Statistical analyses were performed with IBM SPSS statistics 22.0. Continuous variables were summarized as mean ± SD or median (quartiles) and compared using unpaired Student's *t*-test or the Mann–Whitney test. Categorical variables were presented as absolute numbers and percentages and compared by the chi-square test. Time to conversion to SR was assessed via Kaplan–Meier analysis. We used the log-rank test to compare cumulative progression curves for AF conversion in the two groups. Outcomes were considered statistically significant at *P* level <0.05.

## 3. Results

From February 2016 to December 2016, 41 consecutive patients were considered eligible for this study based on our inclusion criteria and were consequently randomly allocated to either the AMIO group (*n*=21) or the AMIO + WXKL group (*n*=20). These two groups were similar in terms of demographic, clinical, and echocardiographic characteristics, as summarized in Tables 1 and 2. Most patients in both groups exhibited recent-onset AF with arterial hypertension. However, there were more patients with type 2 diabetes mellitus in the AMIO + WXKL group compared with the AMIO monotherapy group (*P*=0.03). QTc interval prior to administration was longer in the AMIO + WXKL group than in the AMIO group (456 ± 25 ms vs. 437 ± 29 ms, *P*=0.03). The mean heart rate of the patients following conversion showed no significant difference in the AMIO group than in the AMIO + WXKL group (67.4 ± 14.1 beats per minutes vs. 62.3 ± 9.2, *P*=0.24). The echocardiographic parameters, including LAD, LVEDD, LVESD, IVS, and LVEF, did not differ significantly between the two groups. None of the patients in either group had previously undergone cardiac surgery.

### 3.1. Effects of Amiodarone Plus Wenxin Keli

**Figure 1:**
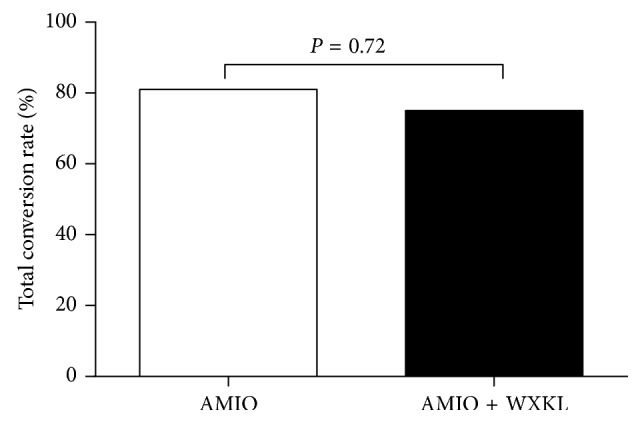
Effect of the combination of AMIO plus WXKL (*n*=20) versus AMIO monotherapy (*n*=21) on conversion rate.

**Figure 2:**
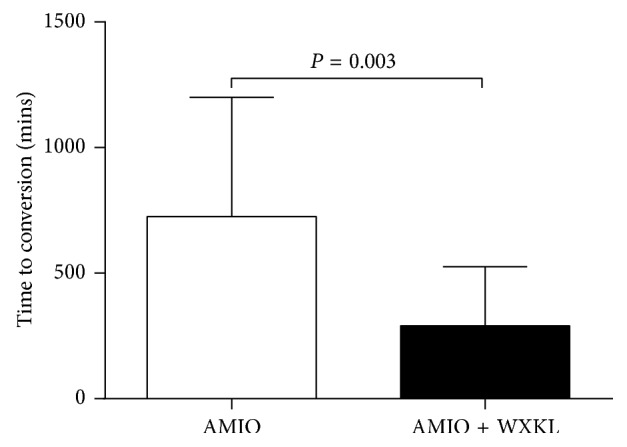
Effect of the combination of AMIO plus WXKL (*n*=20) versus AMIO monotherapy (*n*=21) on conversion time.

**Figure 3:**
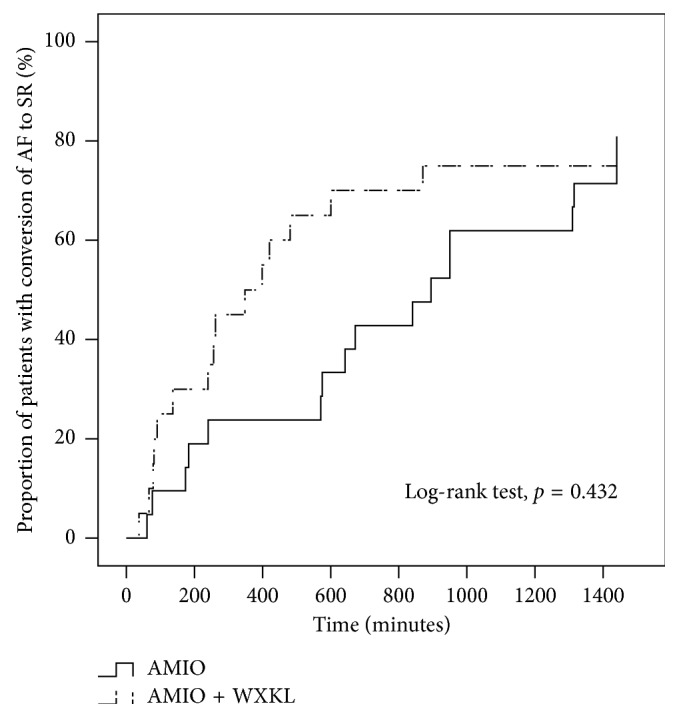
Cumulative progression of atrial fibrillation conversion to sinus rhythm in the AMIO plus WXKL group (*n*=20) versus the AMIO monotherapy group (*n*=21) during the initial 24 hours of treatment.

The proportion of patients converted to sinus rhythm within 24 hours in the AMIO group was not significantly different from that of the AMIO + WXKL group (81.0% vs. 75.0%, *P*=0.72) (Figure 1). In the AMIO group, there was no significant difference in the conversion rate (83.3% vs. 80.0%, *P* > 0.99) or conversion time (808 ± 447 vs. 691 ± 501 minutes, *P*=0.66) between patients presenting with first episodes of AF and those with recurrent AF episodes. Similarly, in the AMIO + WXKL group, no significant differences in the conversion rate (66.7% vs. 76.5%, *P* > 0.99) but marked differences in the conversion time (646 ± 319 minutes vs. 236 ± 178 minutes, *P*=0.02) were observed. In the AMIO group, the conversion rate within 24 hours for male patients had a tendency to be higher than that of female patients (100.0% vs. 63.6%, *P*=0.09). Comparatively, in the AMIO + WXKL group, these proportions were 100.0% and 58.3%, respectively (*P*=0.06).  Figure 2 shows that the secondary endpoint, conversion time, was markedly shortened in the AMIO + WXKL group compared with the AMIO-only group (291 ± 235 minutes vs. 725 ± 475 minutes, *P*=0.003). Cumulative conversion progression in the two groups is shown in Figure 3.

### 3.2. Electrocardiography

Baseline QTc interval was significantly different in the AMIO and AMIO + WXKL groups (437 ± 29 ms vs. 456 ± 25 ms, *P*=0.03). The QTc interval at the time of conversion was not significantly different between the AMIO and AMIO + WXKL groups (446 ± 34 ms vs. 439 ± 34 ms, *P*=0.53). The QTc interval tended to be increased nonsignificantly in the AMIO group (437 ± 29 ms to 446 ± 34 ms, *P*=0.26), but was significantly shortened in the AMIO + WXKL group (456 ± 25 ms to 439 ± 34 ms, *P*=0.002). The PR interval after conversion was not significantly different in the AMIO + WXKL group from the AMIO group (159 ± 33 ms vs. 145 ± 29 ms, *P*=0.31). No serious adverse events, including substantial QTc prolongation (defined as QTc interval >550 ms), were observed during the 24 h in either group.

## 4. Discussion

The main finding of this randomized controlled trial is that the combination of Wenxin Keli (18 g three times daily) and amiodarone (loading dose 5 mg/kg in 1 hour followed by 50 mg/h) was safe and superior to amiodarone monotherapy in chemical conversion of recent-onset AF. Conversion time to restore sinus rhythm was markedly shortened in patients receiving the combination therapy (291 ± 235 minutes) compared with amiodarone alone (725 ± 475 minutes) despite similar results for conversion rate within 24 hours between the two groups (75.0% vs. 81.0%, respectively). In addition, the QTc interval shortened from baseline to 24 hours in the combination group (456 ± 25 ms to 439 ± 34 ms), with an opposite result in the amiodarone monotherapy group (437 ± 29 ms to 446 ± 34 ms). No serious adverse effects occurred during the study. The females had a tendency to be older than the males in both the AMIO group (75 ± 9 years vs. 68 ± 16 years, *P*=0.22) and the AMIO + WXKL group (73 ± 12 years vs. 68 ± 10 years, *P*=0.37). Furthermore, the mean LAD in the females was larger with no significant difference than that in the males in both the AMIO group (40.9 ± 6.6 mm vs. 40.1 ± 5.4 mm, *P*=0.79) and the AMIO + WXKL group (41.6 ± 5.8 mm vs. 39.8 ± 5.3 mm, *P*=0.49). To the best of our knowledge, the age and the LAD are the independent risk factors of AF, which may explain the lower conversion rates of the females of older ages with larger LADs than the males in the two groups.

Wenxin Keli, a traditional Chinese medicine composed of Nardostachys, Codonopsis, Notoginseng, Amber, and Rhizoma, is a formally approved drug that has proven effective and safe for treatment of several forms of cardiac arrhythmia, such as atrial fibrillation and premature ventricular contractions [8, 9]. In isolated rabbit left ventricular myocyte models, Wenxin Keli preferentially inhibited late sodium current while the QRS duration remained less affected at different pacing rates. At higher concentrations (>1 mg/mL), this drug also prevented QT prolongation, suppressed early and delayed postdepolarizations, and subsequent triggered activity with no significant effects on the L-type calcium current (*I*_Ca,L_) and gave a positive staircase pattern in contractility [10]. This is a possible explanation as to why we found a significant decrease in QTc interval at the end of the study compared with the QTc at baseline in the Wenxin Keli plus amiodarone group. Wenxin Keli attenuated ischemia-induced ventricular arrhythmias in rats *in vivo* by significantly reducing the amplitude of *I*_Ca,L_ via decreasing the rate of activation, while also inhibiting the transient outward potassium current (*I*_to_) by increasing the rate of deactivation [11].

According to the results from a prospective, randomized study on paroxysmal atrial fibrillation (PAF) due to hyperthyroidism, sinus rhythm was observed in 91.2% of patients with Wenxin Keli and 89.9% of patients with sotalol after three months [12]. This suggests equal efficacies of both drugs in sinus rhythm conversion. The rate of maintenance of sinus rhythm in the Wenxin Keli-Western medicine group (84.6%) exceeded that of the Western medicine group (62.7%), revealing a significant beneficial effect in the combination group. Despite inadequate data to definitively indicate the efficacy of Wenxin Keli on PAF, this meta-analysis provides encouraging evidence for the effects of Wenxin Keli on sinus rhythm [4]. Our results showed that there was no significant difference in the conversion rate within 24 hours between the AMIO group and the combination group, but this does not necessarily contradict the aforementioned meta-analysis. This is because, to the best of our knowledge, diabetes mellitus is an independent risk factor for AF. In a diabetic rabbit model, high glucose and H_2_O_2_ stimulation promoted atrial fibroblast proliferation [13]. In some cases, atrial fibrillation is considered a complication of hypoglycemia [14, 15]. As such, due to numerous metabolic abnormalities, atrial structural, electrical, and electromechanical remodeling take place [16]. In the combination group, there were more diabetic patients than in the AMIO monotherapy group. As a result, the conversion rate of the combination group may be greatly reduced. Studies of larger sample sizes are therefore warranted to eliminate confounding factors, such as diabetes mellitus.

Amiodarone is widely used for conversion in patients with abnormal left ventricular function or ischemia, but it is limited by its delayed onset of action and lower conversion rate compared with other antiarrhythmic agents. Wenxin Keli is a type of Chinese medicine composed of five different components: Nardostachys jatamansi DC (Gansong), Radix Notoginseng (Sanqi), Succinum (Hupo), Polygonatum sibiricum (Huangjing), and Codonopsis pilosula (Dangshen) [7, 17–20]. Previous experiments have demonstrated the effects of these components on ion channel function in the atria. WXKL shortens atrial action potential durations and blocks the sodium channels [3]. It shifts the steady-state availability of sodium channels to more negative potentials in atrial than in ventricular cells in a dose-dependent manner [6]. These would be expected to prolong atrial effective refractory periods and induce postrepolarization refractoriness. The consequence would be a reduction in the critical interval for reexcitation given by APD-ERP [21], reflecting a lower likelihood of reentry [3, 22]. Our findings are in keeping with previous clinical studies reporting the similar efficacy between Wenxin Keli and sotalol for rhythm control in paroxysmal AF associated with hyperthyroidism [12]. In a prospective randomized pilot study, addition of a single dose of ranolazine, an anti-anginal and anti-ischemic agent with atrial-selective inhibition of late sodium channel current, substantially increased AF conversion rate at 24 hours by 23%, in addition to significantly accelerating SR restoration by >4 hours compared with amiodarone treatment [23]. In another study, the efficacy benefit of ranolazine plus amiodarone was more accentuated in patients with left atrial (LA) enlargement (>46 mm) determined by transthoracic echocardiography, whereas amiodarone alone was as highly efficient as combination therapy in patients with smaller LA size [24]. Moreover, QTc was not one of the matching variables as QTc interval has not been widely accepted to be an independent risk factor for AF or AF conversion but we cannot exclude that this could have modified the risk of incident AF or the substrate of AF. There was modest QT prolongation in both groups. However, in our study, the QTc interval shortened from baseline to 24 hours in the combination group, which may be due to the synergistic effect of Wenxin Keli [10]. In another prospective, randomized clinical trial, mean time of conversion was significantly shorter in the ranolazine-amiodarone group, suggesting a superior antiarrhythmic effect against postcoronary artery bypass graft (CABG) AF compared to amiodarone alone [25, 26].

An important limitation in our study lies in the small sample size restricted to a single center. This was in part due to the cost of running the trial. Nevertheless, we were able to demonstrate statistical significance for the effects of Wenxin Keli in reducing conversion time. However, the study was underpowered in detecting the difference in the conversion at 24 hours or meaningful changes in the electrocardiographic parameters. Furthermore, the QTc before onset of atrial fibrillation was not included in the study. In our study, effects of WXKL on ventricular conduction and side effects or interactions with our drugs administrated can not be excluded, and there exist no sufficient data supporting atrial selectivity on *I*_Na_+ exclusively. Although our result of shortened QTc with Wenxin Keli-amiodarone therapy proves intriguing and safe compared with trials with other drug combinations in terms of outcomes, confirmation from larger studies is required prior to clinical application.

In conclusion, addition of Wenxin Keli to amiodarone markedly shortened conversion time as well as the QTc interval, despite similar results for conversion rates within 24 hours, with no adverse reactions or proarrhythmic effects. A comparison using a larger sample size with varied echocardiographic parameters in the presence of underlying heart conditions would be merited to confirm the superiority of combination therapy in conversion of recent-onset AF. In this study, we explored the acute effects of WXKL as an adjunct therapy to amiodarone. In future studies, the next step will be to explore whether WXKL is effective in converting AF into sinus rhythm beyond 24 hours.

## Figures and Tables

**Table 1:** Demographics and clinical characteristics of enrolled patients.

Variables	AMIO group (*n*=21)	AMIO + WXKL group (*n*=20)	*P* value
Male	10 (47.6%)	8 (40.0%)	0.62
Age (years)	72 ± 13	71 ± 12	0.73
Smoke	5 (23.8%)	5 (25.0%)	0.93
New-onset/recurrent AF	6/15	3/17	0.29
Onset of AF (hours)^*∗*^	4.00 (0.50–29.50)	2.75 (0.31–13.75)	0.72
HTN	14 (66.7%)	15 (75%)	0.56
T2DM	3 (14.3%)	9 (45.0%)	0.03
Medications			
ACEI/ARBs	12 (57.1%)	14 (70.0%)	0.39
*β*-blockers	12 (57.1%)	11 (55.0%)	0.89
CCBs	4 (19.0%)	13 (65.0%)	<0.05
Statins	16 (76.2%)	15 (75.0%)	0.93
Digoxin	1 (4.8%)	5 (25.0%)	0.07

Data are expressed as numbers (percentage) and mean ± standard deviation. AMIO = amiodarone; WXKL = Wenxin Keli; HTN = arterial hypertension; T2DM = type 2 diabetes mellitus; ACEI/ARBs = angiotensin converting enzyme inhibitiors/angiotensin receptor blockers; CCBs = calcium channel blocker. ^*∗*^“Onset of AF” means the time from the onset of AF to the record of AF.

**Table 2:** Clinical parameters, echocardiographic (ECHO), and ECG characteristics of patients after admission.

Variables	AMIO (*n*=21)	AMIO + WXKL (*n*=20)	*P* value
ECHO			
LAD (mm)	40.5 ± 5.9	40.9 ± 5.5	0.85
LVEDD (mm)	46.7 ± 4.0	48.4 ± 4.8	0.21
LVESD (mm)	30.0 ± 5.4	31.4 ± 6.3	0.44
IVS (mm)	9.8 ± 1.6	9.6 ± 2.5	0.75
LVEF (%)	58.1 ± 8.7	61.3 ± 7.3	0.22

ECG			
QTc_1_ (ms)	437 ± 29	456 ± 25	0.03
QTc_2_ (ms)	446 ± 34	439 ± 34	0.53
*P* value^*∗*^	0.26	0.002^*∗*^	

Clinical parameters			
K+ (mmol/L)	4.0 ± 0.4	4.1 ± 0.5	0.60
Na+ (mmol/L)	142.4 ± 5.0	141.5 ± 3.7	0.51
Cl− (mmol/L)	104.4 ± 3.8	104.1 ± 4.7	0.84
Cr (*μ*mol/L)	69.7 (60.5–79.4)	75.0 (63.6–105.3)	0.19
UA (*μ*mol/L)	327.4 ± 97.4	344.9 ± 105.5	0.58
BUN (mmol/L)	6.4 ± 2.0	7.1 ± 2.7	0.35

Data are expressed as mean ± standard deviation or median (*P*_25_–*P*_75_). QTc_1_ means the QTc interval at baseline. QTc_2_ represents the QTc interval at the end of the study. ^*∗*^*P* value at the end of the study compared with prior to drug administration in the AMIO group and the AMIO + WXKL group, respectively. AMIO = amiodarone; WXKL = Wenxin Keli; LAD = left atrial diameter; LVEDD = left ventricular end diastolic diameter; LVESD = left ventricular end systolic diameter; IVS = interventricular septum; LVEF = left ventricular ejection fraction; HR = heart rate; QTc = heart rate corrected QT interval; K+ = serum potassium; Na+ = serum sodium; Cl− = serum chlorine; Cr = serum creatinine; UA = blood uric acid; BUN = blood urea nitrogen.

## Data Availability

The raw data used to support the findings of this study are available from the corresponding author upon request.
